# Global epidemiology of Duchenne muscular dystrophy: an updated systematic review and meta-analysis

**DOI:** 10.1186/s13023-020-01430-8

**Published:** 2020-06-05

**Authors:** Salvatore Crisafulli, Janet Sultana, Andrea Fontana, Francesco Salvo, Sonia Messina, Gianluca Trifirò

**Affiliations:** 1 Department of Biomedical and Dental Sciences and Morphofunctional Imaging, G. Martino Hospital/University of Messina, Building G, 1, Via Consolare Valeria, 98125 Messina, Italy; 2 Unit of Biostatistics, Fondazione IRCCS Casa Sollievo della Sofferenza, San Giovanni Rotondo, Italy; 3 Inserm UMR 1219, Pharmacoepidemiology Team, Université de Bordeaux, Bordeaux, France; 4 Department of Clinical and Experimental Medicine, University of Messina, Messina, Italy; 5 NEuroMuscularOmnicenter, NEMO-SUD, University Hospital “G. Martino”, Messina, Italy

**Keywords:** Duchenne muscular dystrophy, Epidemiology, Prevalence, Birth prevalence, Systematic review, Meta-analysis

## Abstract

**Background:**

Duchenne Muscular Dystrophy (DMD) is a rare disorder caused by mutations in the dystrophin gene. A recent systematic review and meta-analysis of global DMD epidemiology is not available. This study aimed to estimate the global overall and birth prevalence of DMD through an updated systematic review of the literature.

**Methods:**

MEDLINE and EMBASE databases were searched for original research articles on the epidemiology of DMD from inception until 1st October 2019. Studies were included if they were original observational research articles written in English, reporting DMD prevalence and/or incidence along with the number of individuals of the underlying population. The quality of the studies was assessed using a STrengthening the Reporting of OBservational studies in Epidemiology (STROBE) checklist adapted for observational studies on rare diseases. To derive the pooled epidemiological prevalence estimates, a meta-analysis was performed using random-effects logistic models for overall and birth prevalence and within two different underlying populations (i.e. all individuals and in males only), separately. Heterogeneity was assessed using Cochran’s Q-test along with its derived measure of inconsistency I^2^.

**Results:**

A total of 44 studies reporting the global epidemiology of DMD were included in the systematic review and only 40 were included in the meta-analysis. The pooled global DMD prevalence was 7.1 cases (95% CI: 5.0–10.1) per 100,000 males and 2.8 cases (95% CI: 1.6–4.6) per 100,000 in the general population, while the pooled global DMD birth prevalence was 19.8 (95% CI:16.6–23.6) per 100,000 live male births. A very high between-study heterogeneity was found for each epidemiological outcome and for all underlying populations (I^2^ > 90%). The test for funnel plot asymmetry suggested the absence of publication bias. Of the 44 studies included in this systematic review, 36 (81.8%) were assessed as being of medium and 8 (18.2%) of low quality, while no study was assessed as being of high quality.

**Conclusions:**

Generating epidemiological evidence on DMD is fundamental to support public health decision-making. The high heterogeneity and the lack of high quality studies highlights the need to conduct better quality studies on rare diseases.

## Background

Duchenne Muscular Dystrophy (DMD) is a rare neuromuscular X-linked disorder that belongs to a group of disorders known as dystrophinopathies. DMD is caused by mutations in the dystrophin gene that lead to the absence of dystrophin or structural defects of this protein. The lack of functional dystrophin in turn impairs the structure and function of myofibres which are essential for physiological growth of muscle tissue [1]. Due to the localization of the dystrophin gene on the X chromosome, DMD predominantly affects male children, while females are likely to be asymptomatic “healthy carriers” [2].

DMD is characterized by a progressive degeneration of skeletal muscles, with symptoms that manifest early, at around 3 years, causing loss of ambulation within the 13 year of life, followed by cardiac complications (e.g. dilated cardiomyopathy and arrhythmia) and respiratory disorders, including chronic respiratory failure [3]. In the first phase of the disease, the child experiences difficulty in running, climbing stairs, jumping, getting up from the ground, falls frequently and develops a wadding gait with a positive “Gowers’ sign” [1]. The subsequent impairment of the cardiac and respiratory systems is the main cause of death for these patients. Survival is linked to cardiac involvement and has greatly improved thanks to the use of nocturnal ventilation and spinal surgery, with 30% patients surviving beyond 30 years of age [4] and a median survival improved to 30 years [5]. A proportion of DMD patients also experience behavioral and cognitive impairment with intellectual disability, attention hyperactivity disorder (ADHD) and autism spectrum disorders [6]. The disease burden and economic costs are very high and dramatically increase with disease progression [7]. The different burden of comorbidity and mortality in DMD and resulting healthcare utilization patterns compared to the general population highlight the importance of studying DMD populations in detail. The epidemiology of DMD is expected to be generally similar globally, because there is no specific population with a known higher risk. However, variations may arise because of differences in study design and quality. As a result, pooled epidemiological estimates may be considered much more robust and reliable than estimates from single studies. Generating such epidemiological evidence on rare diseases like DMD is fundamental to evaluate the population impact of the disease in terms of burden of disease, to identify unmet clinical needs and to identify eligible target populations for drugs prior to their being marketed. The latter role of epidemiologic research is highlighted in the case of DMD since there are currently only two drugs specifically licensed for the treatment of DMD patients. Specifically, ataluren is licensed in Europe for the treatment of DMD patients with nonsense mutations, (approximately 10–15% of DMD cases) [8], while eteplirsen is licensed in the United States for the treatment of DMD patients who have a confirmed mutation that is amenable to exon 51 skipping (approximately 13% of DMD cases) [9]. This has an important impact on regulatory decisions including the decision to market a drug or not and important cost considerations such as whether a healthcare system is willing to pay for the drug or the adoption of managed entry agreements [10].

In the last 5 years, one narrative and two systematic literature reviews have summarized the global epidemiological evidence on muscular dystrophies [7, 11, 12]. In a recent review, evidence gaps have been highlighted particularly in prevalence and mortality [7] and the economic impact of this disease on healthcare systems is very high due to the needed multidisciplinary care and increases with disease progression, it is crucial to gather updated information on its prevalence, in order to ensure that resources and appropriate services are available for DMD patients world-wide. Moreover, the reviews only included studies up to 2015. This highlights the need to fill the four-year gap to provide updated information. Moreover, previous DMD epidemiology systematic reviews have pooled epidemiological data on DMD, but none of them have in addition performed the quality assessment of the included studies. In general, it is difficult to interpret the results of a study without evaluating its quality and this holds true specifically in rare diseases. The lack of an updated systematic review and meta-analysis which also evaluates study quality in order to aid the interpretation of the meta-analysis itself emerged clearly [11]. The aim of this study is therefore to update the previous systematic review and meta-analysis and to provide a quality assessment of the available epidemiological studies.

## Methods

### Literature search strategy and selection criteria

This systematic review and meta-analysis was carried out in accordance with the Preferred Reporting Items for Systematic Reviews and Meta-Analyses (PRISMA) statement [13], the completed checklist can be found in the Additional file 1. The bibliographic databases MEDLINE and EMBASE were searched individually by two authors (SC, JS) for literature on the epidemiology of DMD from inception until the 1st October 2019. Both databases were searched for terms related to DMD, incidence, prevalence and epidemiology. Citations, titles and abstracts were exported into Endnote X9. The detailed literature search strategy for different databases is provided in Additional file 2.

Only original observational research articles which reported a numerical and well-defined measure of DMD occurrence, such as prevalence, birth prevalence and/or incidence of DMD and were written in English were included. No geographic exclusion criteria were imposed. Narrative or systematic reviews, meta-analyses, book chapters, editorials, personal opinions and conference abstracts were not included; however, the reference lists in reviews and meta-analyses were screened to potentially identify further studies to include. Studies were also excluded if they did not report the year in which the measure of occurrence was estimated. Information on the following items was collected: data source, study population, study years, study design, DMD outcomes description and measure of occurrence. Studies based on segregation analyses were not considered eligible for inclusion. Segregation analyses are methods that use statistical models to propose different hypotheses on the manner of biological inheritance, especially as a function on environmental factors. The epidemiological measures of frequency identified by these studies are therefore generally *predicted*, rather than actual, incidence or prevalence [14]. Only studies reporting the number of DMD cases as well as the underlying population were included in this analysis. If a study presented more than one estimate, the most recent one was used.

After removing duplicates from the two different databases, two review authors (SC, JS) individually screened the titles and abstracts of all records identified to remove articles that were clearly irrelevant; full text articles were then examined to determine whether they met the criteria for inclusion in the review. Any divergences were resolved through discussion or the intervention of a third review author (GT).

### Data extraction and quality of study reporting assessment

Data were individually extracted from the included articles by two authors (SC and JS). The collected information included author(s), year of publication, study catchment area (i.e. geographic zone), data source (i.e. administrative databases, hospital and clinics medical reviews, surveys and other registries), study population (i.e. all living individual, patients and newborns), study period (i.e. the calendar years at which prevalence was measured), study design (i.e. cross-sectional, survey, prospective and retrospective cohorts or chart-review), DMD definition (i.e. ascertained by clinical examination, muscle biopsy and genetic screening) and the epidemiological estimate, i.e. the main outcome. All measures of DMD epidemiology identified in the articles were classified as either (overall) prevalence and birth prevalence. Prevalence was defined as the number of DMD cases identified at any time, including newly and non-newly diagnosed cases, in a source population potentially at risk prior to birth (i.e. all living persons in a well-defined catchment area), irrespective of age. Birth prevalence was defined as the number of DMD cases identified at birth, including only newly-diagnosed cases, in a source population potentially at risk prior to birth (i.e. all live births in the catchment area) [15]. Prevalence was calculated as the number of DMD cases divided by the individuals underlying the source population (and was multiplied by 100,000) and was distinguished between “point prevalence”, if estimated at a specific calendar year (i.e. the last study period year), and as “period prevalence” if estimated during the whole study period. Studies purporting to measure incidence were considered to constitute birth prevalence because this term is more fitting in the case of congenital anomalies, since the occurrence of congenital defects is often evaluated as a cumulative risk (e.g. number of events per 1000 persons), not as a rate of event occurrence per person-time among healthy individuals [16]. Such studies could include genetic screening at birth or other similar evaluations carried out on newborns and are likely to have higher epidemiological estimates compared to evaluations carried out later in life, as some patients may not have survived adulthood. These studies were therefore considered separately. The quality of study reporting was independently assessed by two reviewers (SC, JS) using a checklist adapted from STrengthening the Reporting of OBservational studies in Epidemiology (STROBE) specifically for observational studies concerning rare diseases epidemiology [17].

Study quality was as classified as low, medium or high concerning the following five fields: description of study design and setting, description of eligibility criteria, study population, description of outcomes and description of the study participants. An overall score of low, medium and high was then assigned to each study. The full algorithm used to assign study quality is found in Additional file 3. Disagreements in score assignments were resolved through discussion or the intervention of a third review author (GT).

### Statistical analysis

For each included study, the overall and birth prevalence of DMD per 100,000 individuals was considered as the primary outcome for the meta-analysis. All statistical analyses were performed on the logit-transformed prevalence (i.e. the logarithm of the prevalence divided by its complement) estimated within each study. Variance and its standard error (SE) were estimated applying the Delta-method on the normal approximation of the distribution of such transformed estimate [18]. The lower and upper bounds of each corresponding 95% confidence interval (95% CI) were calculated as the logit-transformed prevalence ±1.96 times SE and the 95% CI was further reported in its original scale by back transformation. As a subgroup analysis, the meta-analysis was stratified by study quality.

Between-study heterogeneity of epidemiological estimates was assessed using the Cochran’s Q-test [19] along with its derived measure of inconsistency (I^2^), and was considered to be present when Cochran’s Q-test *p*-value was < 0.10 or I^2^ > 40% [20]. Due to their dependence on the precision of included trials [21], I^2^ was also corroborated by its 95%CI calculated following the Q-profile method [22]. Study-specific outcomes were summarized by fixed-effects or random-effects logistic models, according to the absence or the presence of heterogeneity, respectively. In the latter case, meta-regression analyses were further performed to identify potential sources of heterogeneity (i.e. examining the contribution of different study-level covariates to the overall heterogeneity) and subgroups meta-analysis were also performed if necessary. The following variables were identified as potential sources of heterogeneity for further investigation: study design, the year in which the study started, study duration and the continent where the study was conducted. Examination of sources of heterogeneity was based on the statistical significance (from omnibus Wald-type test) evaluated to the examined variables. Moreover, the proportion of the between-studies variance which was explained by each study-level covariate was computed in terms of R^2^, which is defined as the ratio of the total between-studies variance explained by the study-level covariate to total between-studies variance computed from the random effects MA without the study-level covariate.

To investigate the presence of publication bias (which consists in the selective publication of studies in relation to their findings), a funnel plot showing the individual observed study outcome (on the x-axis) against the corresponding standard error (on the y-axis) was reported for each outcome at issue and the asymmetry of each funnel plot was evaluated by the rank correlation test, as proposed by Begg and Mazumdar [23]. It is generally accepted that when there are fewer than ten studies in a meta-analysis, both meta-regression [20] and test for publication bias [24] should not be considered.

Study-specific prevalence estimates (along with their 95% CI) as well as the overall summary prevalence estimate were graphically represented (in log scale) with a forest plot: for each study, ordered by the publication year, a square was plotted whose center projection corresponded to the study-specific estimate. A diamond was used to plot the overall prevalence, the center of which represents the point estimate whereas the extremes of the summary estimate show the 95% CI.

Two-sided *p*-values< 0.05 were considered for statistical significance. Statistical analyses were performed using SAS Software, Release 9.4 (SAS Institute, Cary, NC, USA) and R Foundation for Statistical Computing (version 3.6, package: metafor).

## Results

### Study selection and characteristics

**Fig. 1:**
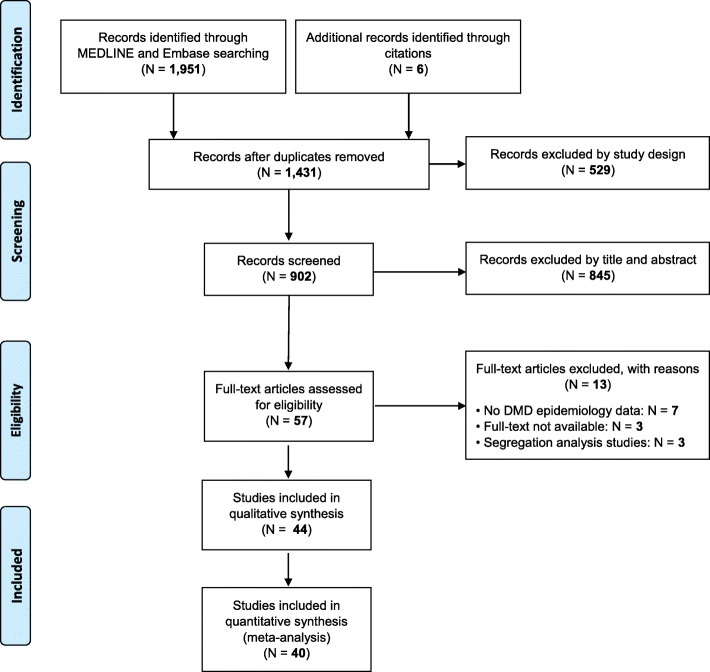
PRISMA flow-chart showing the process of literature search and study selection

**Fig. 2:**
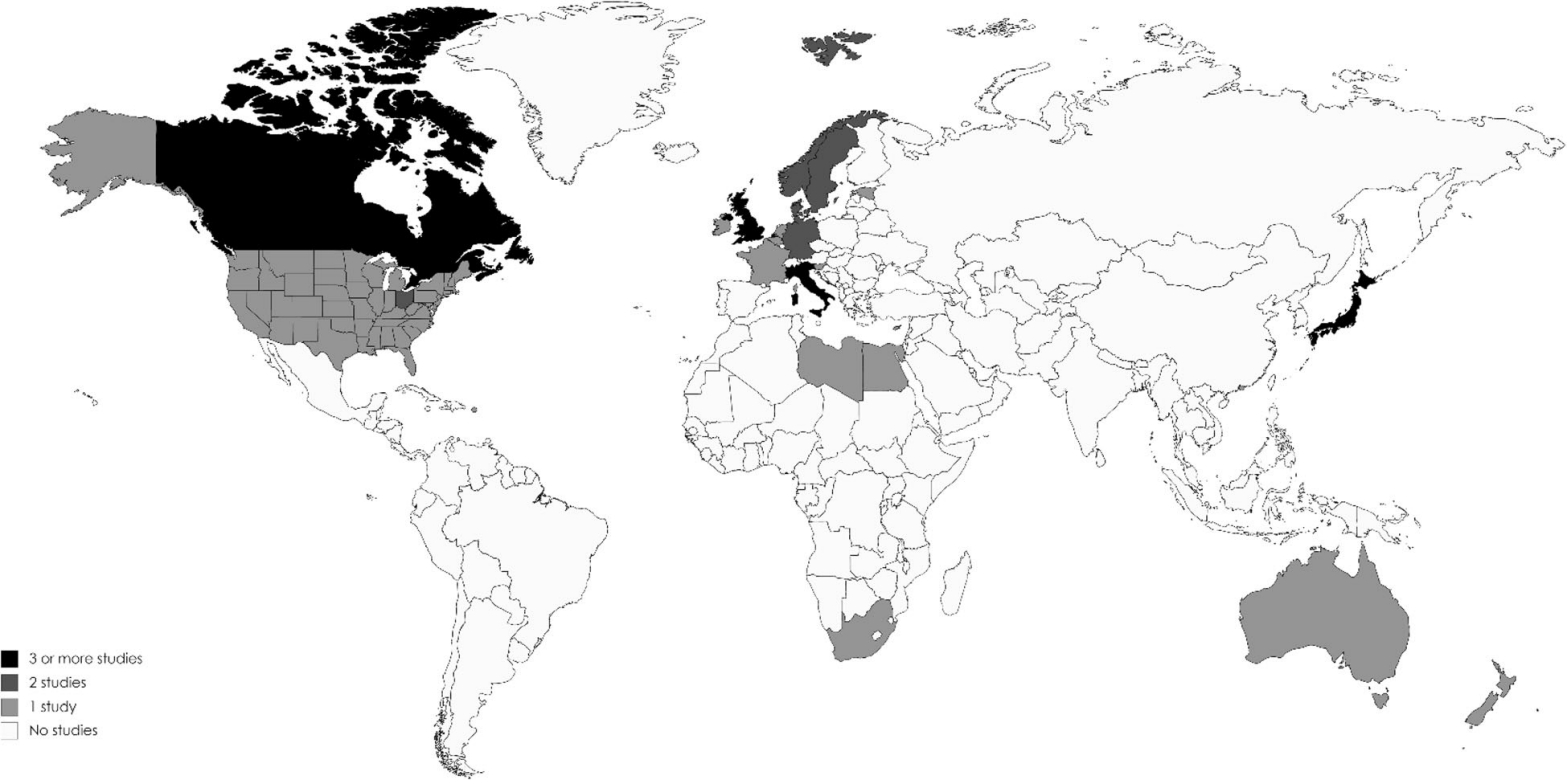
Geographical distribution of the Duchenne muscular dystrophy epidemiological studies included in the systematic review

The flow-chart for study selection is shown in Fig. 1. Overall, the initial literature search identified 1951 studies. Following removal of duplicates (*N* = 520), 1431 abstracts were initially screened and only 57 (4.0%) full-text articles to review were retained for further evaluation. Of these, based on literature review, 44 (77.2%) studies containing information on the global epidemiology of DMD met the eligibility criteria and were therefore included in this systematic review. The detailed characteristics of the included studies are summarized in Table 1. Twenty-two studies (50%) reporting DMD prevalence [25–46] and 29 studies (65.9%) reporting DMD birth prevalence [25–27, 30, 34, 37, 39, 47–68] were included. Six studies (13.6%) reported both DMD prevalence and birth prevalence [25, 26, 30, 34, 37, 39]. The majority of the studies included (*N* = 28; 63.6%) were conducted in Europe. The geographical distribution of the studies included in the review is shown in Fig. 2. The studies conducted by Lefter et al., Norman et al., Leth et al. and Radhakrishnan et al. [27, 28, 46, 60] were excluded from the meta-analysis because the denominator used to calculate the prevalence was not reported in the full-text article.

**Table 1:** Characteristics of the included studies on Duchenne muscular dystrophy epidemiology

Author, Year of publication	Catchment area	Data source	Population	Study years	Study design	DMD definition	Prevalence type	Epidemiological estimate per 100,000 [95% CI]
DMD Prevalence								
Danieli, 1977 [25]	Four districts of Veneto Region (Italy)	Hospital records review	Patients with a diagnosis of DMD from 1952 to 1972	1952–1972	Retrospective chart-review study	High serum CK levels on samples of fresh serum from subjects at rest and 6 h after vigorous physical exercise	Period prevalence per 1000,000 males and females of any age	3.4 [2.8–4.2] per 100,000
Monckton, 1982 [26]	Alberta (Canada)	Hospital/clinic chart review	Cases recorded by three muscular dystrophy association Clinics as well as cases recorded at the Genetics Clinics of the University of Alberta and the Alberta Children’s Hospital	1950–1979	Retrospective chart-review study	–	Point prevalence in 1979 per 100,000 males of any age	9.5 [7.8–11.6] per 100,000
Leth, 1985 [27]	Denmark	Collection of data from hospital departments, nursery homes and general practitioners	445 patients with progressive muscular dystrophy alive January 1st 1965	1965–1975	Retrospective population-based cohort study	Histological changes in muscular tissue, typical electromyographic changes, high serum CK levels, family occurrence of progressive muscular dystrophy	Point prevalence per 1000,000 in 1965	6.94 per 100,000^a^
Radhakrishnan, 1987 [28]	Benghazi, Lybia	Hospital records review	Patients resident of Benghazi with neuromuscular disorders over the period January 1983–1985	1983–1985	Retrospective chart-review study	DMD diagnosis based on clinical examination, family history, serum CPK, electromyography and investigations to exclude acquired disorders	Point prevalence in 1985 per 100,000	6.0 per 100,000^a^
Nakagawa, 1991 [29]	Okinawa (Japan)	Hospital chart review (data collected from hospital departments, nursing homes and social health centers)	Patients with DMD in the whole Okinawa prefecture	1989	Retrospective population-based cohort study	Clinical presentation, high serum CK levels, electromyography, lens examination and immunohistochemical studies with antidystrophin antibody	Point prevalence per 100,000 males of any age	7.1 [5.16–9.6] per 100,000 males
van Essen, 1992 [30]	The Netherlands	Linkage database containing Dutch DMD registry, National Medical Registration file, Death Registry, Medical Genetics database	All living DMD patients on January 1, 1983 in the Netherlands	1961–1982	Retrospective population-based cohort study	A score was given considering the clinical status, serum CK levels, electromyograms, muscle biopsy findings, electrocardiograms, and familial occurrence compatible with X-linked recessive inheritance, together with the CK levels in the mother and/or sister when available	Point prevalence in 1983 per 100,000 males of any age	5.4 [4.9–6.0] per 100,000 males
Ahlström, 1993 [31]	Örebro (Sweden)	Clinical chart and administrative data (e.g. early retirement pension, temporary disability pension) review	Patients with a diagnosis of DMD between 1974 and 1987	1974–1988	Retrospective chart-review study	–	Point prevalence in 1988 per 100,000 males and females of any age	0.7 [0.2–2.7] per 100,000
Ballo, 1994 [32]	South Africa	Referrals requested from practitioners and genetic clinics	Patients with a diagnosis of DMD between 1987 and 1992	1987–1992	Observational cohort study using retrospectively data	High serum CK levels, electromyography and genetic testing	Period prevalence per 1000 males of any age	0.9 [0.8–1.1] per 100,000 males
Hughes, 1996 [33]	Northern Ireland	Primary data: Mailed survey Secondary data: hospital/clinic chart review, administrative data, patient registry	Patients with DMD identified from the records of the North Ireland Muscle Clinic, the North Ireland Medical Genetic Department and from general practitioners, physicians and pediatricians data	1993–1994	Epidemiological survey. Population-based cohort study using prospectively and retrospectively collected data	Not specified	Point prevalence in 1994 per 1000,000 males and females of any age	8.2 [6.3–10.4] per 100,000
Hughes, 1996 [33]	Northern Ireland	Primary data: mailed survey Secondary data: hospital/clinic chart review, administrative data, patient registry	Patients with DMD identified from the records of the North Ireland Muscle Clinic, the North Ireland Medical Genetic Department and from general practitioners, physicians and pediatricians data	1993–1994	Epidemiological survey. Population-based cohort study using prospectively and retrospectively collected data	Not specified	Point prevalence in 1994 per 1000,000 males of any age	4.3 [3.3–5.4] per 100,000 males
Peterlin, 1997 [34]	Slovenia	Registries and medical records review	DMD cases diagnosed in the period 1969–1984	1969–1984	Retrospective population-based cohort study	DMD diagnosis based on the clinical picture, serum enzymes, electromyography and muscle biopsy	Point prevalence in 1990 per 100,000 males of any age	2.9 [2.0–4.2] per 100,000 males
Siciliano, 1999 [35]	North-West Tuscany (Italy)	Primary data: mailed survey Secondary data: hospital/clinical records review, administrative databases	Patients treated in the Unit for Muscle Diseases, University of Pisa	1997	Epidemiological survey. Population-based cohort study using prospectively and retrospectively collected data	Genetic testing (genomic DNA analysis and dystrophin analysis), clinical exam, high serum CK levels, family history, muscle biopsy	Point prevalence per 100,000 males and females of any age	1.7 [1.1–2.6] per 100,000
Darin, 2000 [36]	Western Sweden	Residential and outpatient registers, muscle biopsy registries and administrative databases	All individuals with neuromuscular disorders born between 1979 and 1994 and admitted in one of the seven hospitals in the region before 1st January 1995	1995	Retrospective population-based cohort study	Clinical exams, high serum CK levels, family history, muscle biopsy, genetic testing	Point prevalence per 100,000 males, aged less than 16 years	16.8 [11.4–23.8] per 100,000 males
Jeppesen, 2003 [37]	Aarhus (Denmark)	Medical records of all DMD patients in the Institute of Neuromuscular Diseases, Respiratory Centre East at the State University Hospital and Respiratory Centre West at Aarhus University Hospital	Male Danish population in the period January 1, 1977 to January 1, 2002 and Danish newborn males	1977–2002	Retrospective population-based cohort study	Until 1993, ICD-8 code 330.39 (*dystrophia musculorum progressiva*) or subcode 330.38 (*dystrophia musculorum progressiva, typus Duchenne*); from 1994 onward, ICD-10 code G71.0 (*dystrophia musculorum*) or subcode G71.0H (*dystrophia musculorum gravis, Duchenne*)	Point prevalence in 2002 per 100,000 males of any age	5.5 [4.6–6.5] per 100,000 males
Chung, 2003 [38]	Hong Kong	Hospital/clinic chart review from two University teaching hospitals	332 children aged < 19 years at first assessment with neuromuscular diseases confirmed by using electromyography, muscle biopsy, and/or molecular genetic study	1985–2001	Prospective population-based cohort study	High serum CK level, nerve conduction study, electromyography, muscle biopsy, and molecular genetic study of blood DNA	Point prevalence in 2001 per 1000,000 males aged less than 19 years	9.8 [7.7–12.6] per 100,000 males
Talkop, 2003 [39]	Estonia	Hospital/clinic chart review, mailed survey, administrative database, patient registry	All patients with DMD born and diagnosed in the period 1977–1999 in Estonia	1994–1999	Epidemiological survey. Observational cohort study using retrospectively collected data	Not specified	Point prevalence in 1998 per 100,000 males aged less than 20 years	12.8 [8.3–18.8] per 100,000 males
El-Tallawy, 2005 [40]	Assiut (Egypt)	Door-to-door community survey	52,203 subjects, identified from a door-to-door survey	1996–1997	Cross-sectional study	Electrophysiological and biochemical (high serum CK levels) investigations, genetic testing, muscle biopsy	Point prevalence in 1997 per 100,000 males and females of any age	7.7 [2.1–19.6] per 100,000
Norwood, 2009 [41]	Northern England	Database of the Institute of Human Genetics in Newcastle and disease-specific databases	All registered patients (children and adults) with inherited muscle diseases diagnosed and currently seen by the neuromuscular team at the Institute of Human Genetics at Newcastle University	2007	Retrospective population-based cohort study	Genetic testing and genetic investigations (deletion, duplication or point mutation in the DMD gene)	Point prevalence per 100,000 males of any age	8.3 [6.8–9.8] per 100,000 males
Mah, 2011 [42]	Canada	De-identified data consisting of the clinical phenotypes, diagnostic methods, and molecular genetic reports from DBMD patients from the Canadian Pediatric Neuromuscular Group	DBMD patients followed by participating Canadian Pediatric Neuromuscular Group centers	2000–2009	Retrospective population-based cohort study	Clinical phenotypes, diagnostic methods (MLPA, muscular biopsy) and molecular genetic reports	Period prevalence per 10,000 males from birth to 24 years	10.6 [9.7–11.5] per 100,000 males
Rasmussen, 2012 [43]	South-Eastern Norway	Prospectively collected patient data	Patients aged under 18 years treated by neuropediatricians	2005	Prospective population-based cohort study	Genetic testing (sequencing of the dystrophin gene) and/or muscular biopsy	Point prevalence per 100,000 males from birth to 18 years	16.2 [11.5–22.8] per 100,000 males
Romitti, 2015 [44]	USA	MD STAR*net* database	Patients born from January 1982, to December 2011, resided in an MD STARnet site during any part of that time period, and was diagnosed with childhood-onset DBMD	1982–2011	Cross-sectional study	ICD-9 CM code: 359.1 or ICD-10 CM code: G71.0	Point prevalence in 2010 per 10,000 males aged 5–24 years	10.2 [9.2–11.2] per 100,000 males
Ramos, 2016 [45]	Puerto Rico	Data from 141 patients attending the Muscular Dystrophy Association neuromuscular clinics in Puerto Rico (4 clinics in total)	141 patients attending the Muscular Dystrophy Association neuromuscular clinics in Puerto Rico	2012	Retrospective epidemiological survey	“Definite” cases have symptoms referable to DMD and either (1) a documented DMD gene mutation, (2) muscle biopsy evidencing abnormal dystrophin without an alternative explanation, or (3) CK level at least 10 times normal, pedigree compatible with X-linked recessive inheritance, and an affected family member	Point prevalence per 100,000 males of any age	5.2 [4.2–6.4] per 100,000 males
Lefter, 2016 [46]	Republic of Ireland	Demographic, clinical, physiologic, histopathology, serology, and genetic data from retrospectively and prospectively identified patients	Adults (≥18 years old) living in the Republic of Ireland ≥5 years	2012–2013	Population-based study using retrospectively and prospectively collected data	Genetic and electrophysiological tests	Point prevalence in 2013 per 100,000 males (≥18 years old)	3.0 [2.3–3.7] per 100,000 males
DMD Birth prevalence								
Brooks, 1977 [47]	South Eastern Scotland	Survey and clinical records review	All cases of DMD who had been born between 1953 and 1968	1953–1968	Retrospective epidemiological survey	–	Period birth prevalence	26.5 [19.9–35.2] per 100,000 live male births
Danieli, 1977 [25]	Four districts of Veneto Region (Italy)	Hospital records review	Patients with a diagnosis of DMD from 1952 to 1972	1952–1972	Retrospective chart-review study	High serum CK levels on samples of fresh serum from subjects at rest and 6 h after vigorous physical exercise	Period birth prevalence	28.2 [22.1–35.8] per 100,000 live male births
Takeshita, 1977 [48]	Shimane (Japan)	Questionnaires sent to nurse-teachers in infant schools, primary schools and junior high schools in Shimane	–	1956–1970	Epidemiological survey	Neurological exams, electromyography, high CPK levels, muscle biopsy	Period birth prevalence	20.8 [13.3–32.6] per 100,000 live male births
Drummond, 1979 [49]	New Zealand	Prospectively collected patient data	101 consecutive live births at St Helen’s Hospital, Auckland, New Zealand	–	Cross-sectional study	High CPK levels in newborn blood spot	Birth prevalence	20.0 [5.5–72.9] per 100,000 live male births
Cowan, 1980 [50]	Australia	Survey and clinical records review	DMD cases in New South Wales and in the Australian Capital Territory between 160 and 1971	1960–1971	Retrospective epidemiological survey	–	Period birth prevalence	18.6 [15.3–22.6] per 100,000 live male births
Danieli, 1980 [51]	Veneto Region (Italy)	Hospital records review	DMD cases born in the period 1959–1968	1952–1972	Retrospective epidemiological survey	Abnormal CK values	Period birth prevalence	28.2 [23.3–34.2] per 100,000 live male births
Bertolotto, 1981 [52]	Turin (Italy)	Clinical records review	All DMD cases born in Turin between 1955 and 1974.	1955–1974	Retrospective epidemiological survey	High CPK levels and electromyography	Period birth prevalence	24.2 [19.3–30.5] per 100,000 live male births
Monckton, 1982 [26]	Alberta (Canada)	Hospital/clinic chart review	Cases recorded by three muscular dystrophy association Clinics as well as cases recorded at the Genetics Clinics of the University of Alberta and the Alberta Children’s Hospital	1950–1979	Retrospective chart-review study	–	Period birth prevalence	26.2 [21.7–31.5] per 100,000 live male births
Nigro, 1983 [53]	Campania Region (Italy)	Prospectively collected patient data	DMD cases born in Campania from 1960 until 1971	1969–1980	Cross-sectional study	DMD diagnosis based on age of onset of symptoms, age of onset of the chairbound stage, pseudohypertrophy of calf muscle, marked elevation of CPK levels, muscle biopsy	Period birth prevalence	21.7 [18.5–25.3] per 100,000 live male births
Dellamonica, 1983 [54]	France	Prospectively collected patient data	Blood samples of 158,000 newborns obtained 4 to 8 days postnatally	1978	Cross-sectional study	High CPK levels in newborn blood spot	Birth prevalence	16.9 [9.7–29.5] per 100,000 live male births
Leth, 1985 [27]	Denmark	Collection of data from hospital departments, nursery homes and general practitioners	445 patients with progressive muscular dystrophy alive January 1st 1965	1965–1975	Retrospective population-based cohort study	Histological changes in muscular tissue, typical electromyografic changes, high serum CK levels, family occurrence of progressive muscular dystrophy	Period birth prevalence	22.2 per 100,000^a^
Scheuerbrandt, 1986 [55]	West Germany	Prospectively collected patient data	–	1977–1984	Cross-sectional study	High CK activity in newborn blood spot	Period birth prevalence	27.2 [20.5–36.0] per 100,000 live male births
Mostacciuolo, 1987 [56]	Five districts of Veneto Region (Italy)	Hospital records review	DMD cases born in the period 1959–1968	1955–1984	Retrospective epidemiological survey	DMD diagnosis based on electromyography, muscle biopsy, serum enzymes, and clinical history of the patients	Period birth prevalence	26.0 [34.4–53.9] per 100,000 live male births
Takeshita, 1987 [57]	Western Japan	Data collected from the preschool development screening program, from public institutions for children and 5 hospitals	DMD cases born between 1956 and 1980	1956–1980	Retrospective population-based cohort study	DMD diagnosis based on electromyography, serum CK levels and muscle biopsy	Period birth prevalence	19.1 [14.5–25.2] per 100,000 live male births
Greenberg, 1988 [58]	Canada	Prospectively collected patient data	18,000 newborn males screened for DMD in the routine Manitoba perinatal screening program	1986–1987	Cross-sectional study	High CK levels in newborn blood spot, muscle biopsy	Period birth prevalence	27.8 [11.9–65.0] per 100,000 live male births
Tangsrud, 1989 [59]	Southern Norway	Clinical records and national databases review	All boys with a known history of Duchenne muscle dystrophy born during the period 1968–1977	1968–1977	Retrospective chart-review study	Muscle biopsies, electromyographic changes, high serum CK levels	Period birth prevalence	21.9 [13.5–35.6] per 100,000 males
Norman, 1989 [60]	Wales	Retrospectively and prospectively collected patient data	–	1971–1986	Cross-sectional study	High CK levels	Period birth prevalence	24.7 per 100,000 males^a^
van Essen, 1992 [30]	The Netherlands	Linkage database containing Dutch DMD registry, National Medical Registration file, Death Registry, Medical Genetics database	All males with DMD both born and diagnosed in the period 1961–1982 in the Netherlands	1961–1982	Retrospective population-based cohort study	A score was given considering the clinical status, serum CK levels, electromyograms, muscle biopsy findings, electrocardiograms, and familial occurrence compatible with X-linked recessive inheritance, together with the CK levels in the mother and/or sister when available.	Period birth prevalence	23.7 [20.7–26.7] per 100,000 live male births
Merlini, 1992 [61]	Bologna (Italy)	Clinical records review	Children born between 1970 and 1989 in Bologna (Italy)	1970–1982	Retrospective epidemiological survey	–	Period birth prevalence	25.8 [16.7–39.8] per 100,000 live male births
Bradley, 1993 [62]	Wales	Blood samples obtained through screening program for phenylketonuria and congenital hypothyroidism in all maternity units throughout Wales	–	1990–1992	Cross-sectional study	High CK levels in newborn blood spot, genetic testing, molecular genetic mutation analysis, muscle biopsy and dystrophin analysis.	Period birth prevalence	26.3 [13.8–49.9] per 100,000 live male births
Peterlin, 1997 [34]	Slovenia	Registries and medical records review	DMD cases diagnosed in the period 1969–1984	1969–1984	Retrospective population-based cohort study	DMD diagnosis based on the clinical picture, serum enzymes, electromyography and muscle biopsy	Period birth prevalence	13.8 [9.6–19.8] per 100,000 live male births
Drousiotou, 1998 [63]	Cyprus	5170 blood samples obtained through the national screening center for phenylketonuria and congenital hypothyroidism	30,014 newborn males screened for DMD	1992–1997	Cross-sectional study	High CK levels in newborn blood spot	Period birth prevalence	16.7 [7.1–39.0] per 100,000 live male births
Jeppesen, 2003 [37]	Aarhus (Denmark)	Medical records of all DMD patients in the Institute of Neuromuscular Diseases, Respiratory Centre East at the State University Hospital and Respiratory Centre West at Aarhus University Hospital	Danish live born males from 1972 to 2001	1992–1996	Retrospective population-based cohort study	Until 1993, ICD-8 code 330.39 (dystrophia musculorum progressiva) or subcode 330.38 (dystrophia musculorum progressiva, typus Duchenne); from 1994 onward, ICD-10 code G71.0 (dystrophia musculorum) or subcode G71.0H (dystrophia musculorum gravis, Duchenne)	Period birth prevalence	18.8 [12.4–25.2] per 100,000 live male births
Talkop, 2003 [39]	Estonia	Hospital/clinic chart review, mailed survey, administrative database, patient registry	All patients with DMD born and diagnosed in the period 1977–1999 in Estonia	1986–1990	Observational cohort study using retrospectively collected data	Not specified	Period birth prevalence	17.7 [8.8–31.6] per 100,000 live male births
Eyskens, 2006 [64]	Antwerp (Belgium)	Prospectively collected patient data	281,214 newborn males screened for dystrophinopathy	1979–2003	Cross-sectional study	High CK levels in newborn blood spot	Period birth prevalence	18.2 [7.1–39.0] per 100,000 live male births
Dooley, 2010 [65]	Nova Scotia (Canada)	Records of DMD diagnosis from the Pediatric Neurology Division (Dalhousie University) and the IWK Health Centre	All patients with DMD in Nova Scotia	1969–2003	Retrospective population-based cohort study	Muscle biopsy or genetic testing	Period birth prevalence	21.3 [13.8–23.8] per 100,000 live male births
Mendell, 2012 [66]	Ohio (USA)	Prospectively collected patient data	37,649 newborn male subjects screened for DMD	2007–2011	Cross-sectional study	High CK levels in newborn blood spot and genetic testing (MLPA)	Period birth prevalence	15.9 [7.3–34.8] per 100,000 live male births
Moat, 2013 [67]	Wales	Blood spots collected routinely as part of the Wales newborn screening program	343,170 newborn blood spots screened for DMD	1990–2011	Cross-sectional study	High CK levels in newborn blood spot	Period birth prevalence	19.5 [15.4–24.5] per 100,000 live male births
König, 2019 [68]	Germany	Neuromuscular centers, genetic institutes and the German patient registries	Patients with either dystrophinopathies or SMA born between 1995 and 2018.	1995–2018	Retrospective epidemiological study	–	Point birth prevalence	1.5 [0.7–3.3] per 100,000 live male births

*Abbreviations*: *CK* Creatinine kinase, *DBMD* Duchenne/Becker muscular dystrophy, *DMD* Duchenne muscular dystrophy, *ICD-8* International Statistical Classification of Diseases and Related Health Problems, 8th edition, *ICD-9* International Statistical Classification of Diseases and Related Health Problems, 9th edition, *ICD-10* International Statistical Classification of Diseases and Related Health Problems, 9th edition, *MPLA* Multiplex ligation-dependent probe amplification

^a^95% confidence intervals could not be calculated as the crude numbers required to calculate the epidemiological estimate were not provided in the papers

### Quality of study reporting assessment

**Fig. 3:**
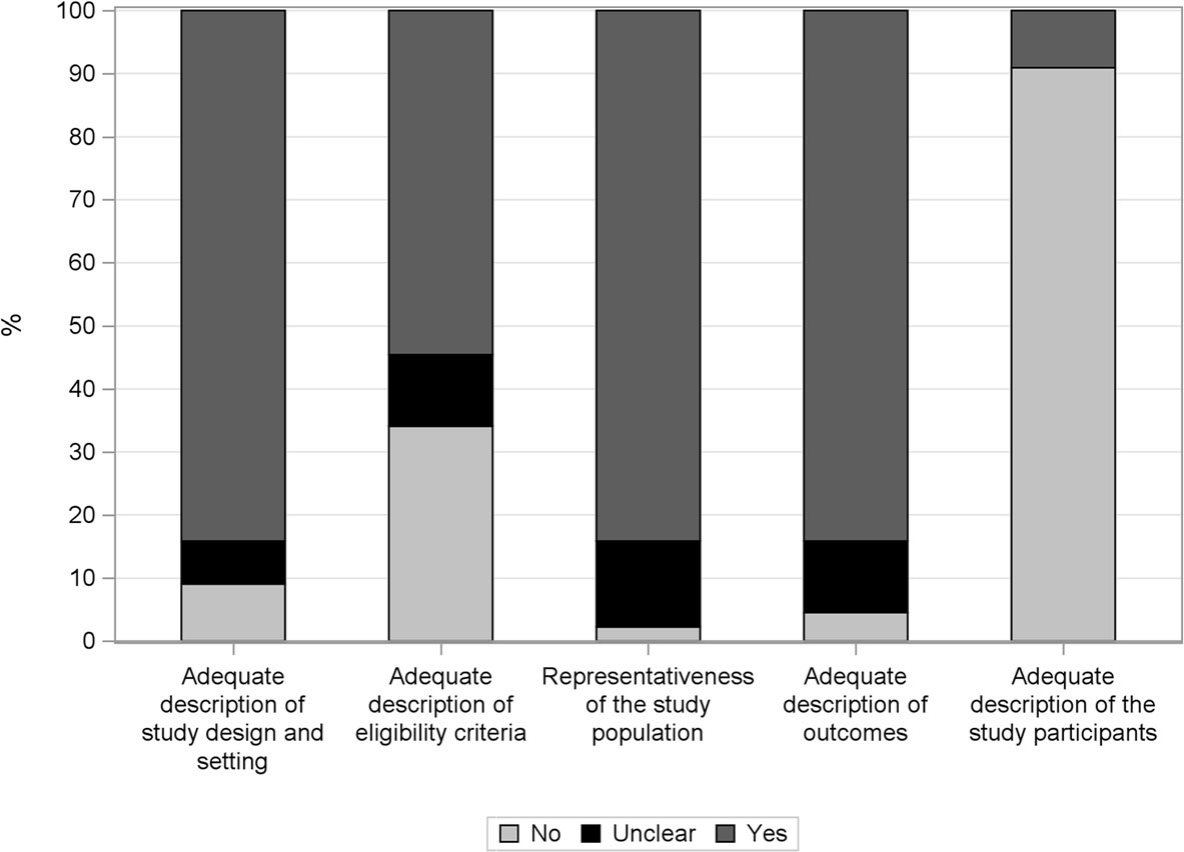
Quality of Duchenne muscular dystrophy epidemiological studies reporting assessment

Overall, the quality of 44 studies was evaluated. In total 36 (81.8%) studies were assessed as being of medium and 8 (18.2%) being of low quality, while no study was assessed as having a high overall quality. Study design and setting were adequately reported in the majority of the studies included in this review (84.1%), while participants were adequately characterized only in 9.1% of the studies. On the contrary, the description of DMD identification was appropriate in 84.1% and unclear in 11.4% of the articles included. Figure 3 summarizes the overall quality of study reporting, which was estimated for all the 44 studies included. More detail about the quality of each included study is reported in Additional file 4.

### Pooled DMD overall and birth prevalence

Of the 22 studies reporting DMD prevalence, 13 (59.1%) were European [25, 27, 30, 31, 33–37, 39, 41, 43, 46], 4 (18.2%) American [26, 42, 44, 45], 2 (9.1%) Asian [29, 38] and 3 (13.6%) were African [32, 40].

**Fig. 4:**
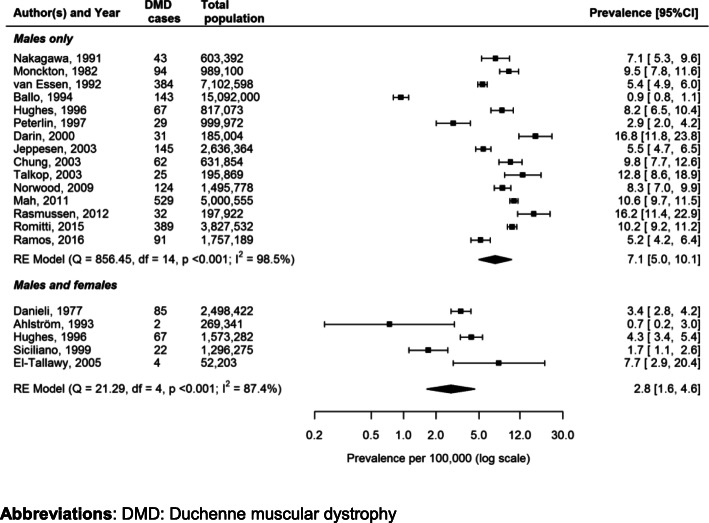
Forest plot of the estimated Duchenne Muscular Dystrophy prevalence per 100,000 cases along with 95% confidence interval in studies which included (in the total population), among male individuals only and the ones which included male and female individuals, separately

The majority of the studies evaluated DMD prevalence through secondary use of data such as clinical charts, administrative databases and patient or disease registries, apart from the study conducted by El-Tallawy et al. [40], which was based on a community survey. The global prevalence of DMD ranged from 0.9 [32] to 16.8 [36] cases per 100,000 males, including a population of neonates to the oldest surviving adults. When considering the general population (i.e. males and females), the global prevalence of DMD ranged from 0.7 to 7.7 cases per 100,000, with the lowest value in Sweden [31] and the highest value in Egypt [40]. The pooled DMD prevalence was 7.1 cases (95% CI: 5.0–10.1) per 100,000 males and 2.8 cases (95% CI: 1.6–4.6) per 100,000 persons (i.e. males and females together) (Fig. 4). A substantial heterogeneity was detected both in males (Cochran’s Q = 856.45, I^2^ = 98.5%, *p* < 0.001) and in the whole population (Cochran’s Q = 21.29, I^2^ = 87.4%, *p* < 0.001). The 95% CI for such I^2^ statistics were 97.2–99.4% and 63.0–99.4%, respectively. However, it is difficult to interpret these findings as heterogeneous on the basis of 5 studies only.

**Fig. 5:**
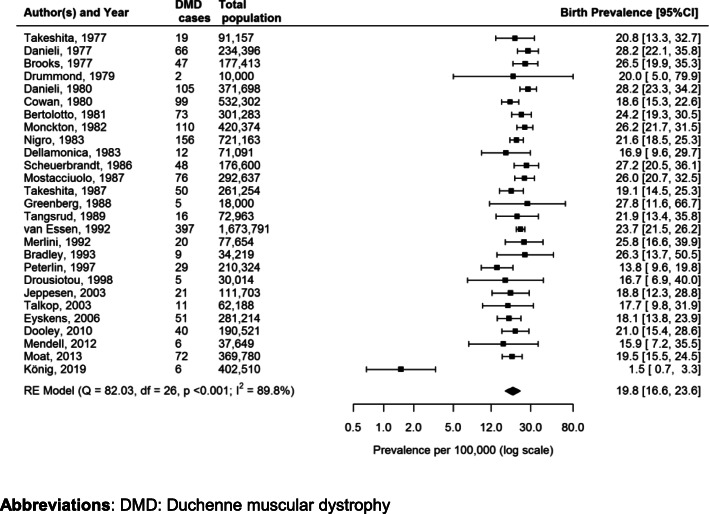
Forest plot of the estimated Duchenne Muscular Dystrophy birth prevalence per 100,000 cases, along with 95% confidence interval

Of the 29 studies reporting DMD birth prevalence, 21 (72.4%) were European [25, 27, 30, 34, 37, 39, 47, 51–56, 59–64, 67, 68], 4 (13.8%) were American [26, 58, 65, 66], 2 (6.9%) were Oceanian [49, 50] and 2 (6.9%) were Asian [48, 57] studies. The global birth prevalence of DMD range was very wide: from 1.5 to 28.2 cases per 100,000 live male births in Germany and Italy, respectively [25, 51, 68]. Eighteen studies (62.1%) [25–27, 30, 34, 37, 39, 47, 48, 50–52, 56, 57, 59, 61, 65, 68] were conducted using secondary data and 11 (37.9%) [49, 53–55, 58, 60, 62–64, 66, 67] using primary data collection, based on questionnaires, blood samples analysis, muscle biopsy and genetic screening. The pooled global birth prevalence was 19.8 cases (95% CI:16.6–23.6) per 100,000 live male births (Fig. 5). A substantial heterogeneity was seen among these studies (Cochrane’s Q = 82.03, I^2^ = 89.8%, *p* < 0.001) with a 95%CI for I^2^ ranging from 75.5 to 95.8%.

The stratification was only possible for medium quality studies (*N* = 36), because there were too few studies of low quality (*N* = 4) and no studies of high quality. The pooled estimate from random effects meta-analysis including all studies with medium quality was 6.8 (4.5–10.2) (I^2^ = 98.4%) and 19.5 (16.3–23.5) (I^2^ = 90.6%) concerning DMD prevalence and birth prevalence, respectively.

**Fig. 6:**
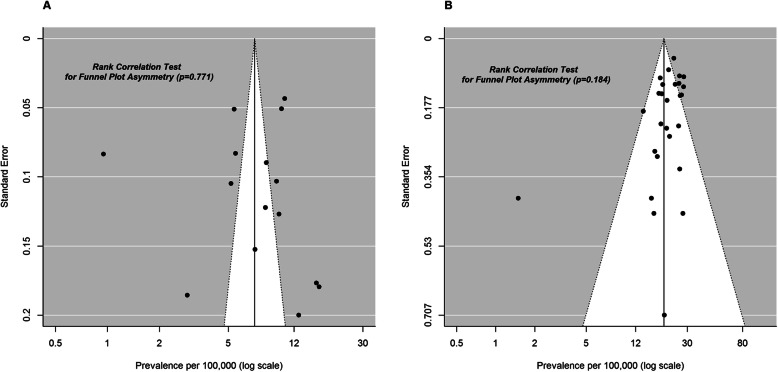
Funnel plots for the estimated Duchenne Muscular Dystrophy (DMD) prevalence in males (panel **a**) and DMD birth prevalence (panel **b**) along with Begg and Mazumdar’s rank correlation test for asymmetry

A visual inspection of the data suggested several outliers, namely Ballo et al. and Peterlin et al. who had very low values for prevalence per 100,000 males while and Darin et al. and Rasmussen et al. had very high values for this same outcome. However, no qualitative differences in study methodology to justify their impact on the pooled estimates were observed. Concerning birth prevalence, König et al. were found to be outliers. This study had problems with data collection in the last study year, as due to privacy issues, DMD cases were under-reported. No publication bias was found based on the funnel plot and Begg and Mazumdar’s rank correlation test for asymmetry both for DMD prevalence and birth prevalence (*p*-values = 0.771 and 0.184, respectively) (Fig. 6).

### Exploration of sources of heterogeneity

In order to explore the sources of heterogeneity of worldwide prevalence estimates, a meta-regression analysis was performed for DMD (males only) and birth DMD outcomes, separately. Meta-regression analysis was not performed in DMD general population because less than 10 studies were available. Since the point prevalence was estimated for almost all DMD studies whereas the period prevalence for almost all birth DMD studies, the information about prevalence type was not considered into the meta-regression analysis.

None of the study level covariates significantly reduced the between-study heterogeneity estimated from the random-effects meta-analysis (i.e. the proportion of explained heterogeneity R^2^ was always lower than 20%) with exception of the “study period” which significantly explained such heterogeneity between DMD birth prevalence study estimates (Wald-type test *p* < 0.001, *R*^2^ = 45%) although a high residual heterogeneity still remained (I^2^ = 83.1, 95%CI = 64.2–94.9%) (Table 2).

**Table 2:** Results of meta-regression analysis Duchenne Muscular Dystrophy (DMD) prevalence and birth prevalence

Outcome	Subgroup	Study-level covariate(s) included into the meta-regression		Heterogeneity assessment				
		Covariate(s) selected	***p***-value*	Cochran’s Q (df)	***p***-value (Q test)	I^**2**^ (%)	Between-study variance^**e**^	***R***^**2**^ (%)^f^
DMD prevalence	Males only (15 studies)	None (random-effects MA)	–	856.4531 (df = 14)	< 0.0001	98.46%	0.4741	–
		Continent^a^	0.2027	450.7452 (df = 12)	< 0.0001	97.90%	0.3857	18.65%
		Study year (begin) + Study duration	0.4195	632.8951 (df = 12)	< 0.0001	97.89%	0.4227	10.84%
		Study design^c^	0.6429	572.9228 (df = 12)	< 0.0001	98.40%	0.4452	6.10%
DMD birth prevalence	All (27 studies)	None (random-effects MA)	–	82.0309 (df = 26)	< 0.0001	89.79%	0.1646	–
		Continent^b^	0.9308	74.7046 (df = 23)	< 0.0001	88.90%	0.1619	1.64%
		Study year (begin) + Study duration	0.0012	60.8329 (df = 23)	< 0.0001	83.13%	0.0905	45.02%
		Study design^d^	0.3189	78.8714 (df = 24)	< 0.0001	87.51%	0.1483	9.90%

*MA* Meta-analysis, *I*^*2*^ Measure of inconsistency, *df* Degrees of freedom referred to the Cochran’s Q test

*P-values from omnibus Wald-type test of parameters (i.e. study-level covariates included into the model)

^a^Continents were regrouped as follows: America North (US and Canada) (4 studies), Europe North/Centre/East (8 studies), Others (Asia East and Africa South) (3 studies)

^b^Continents were regrouped as follows: America North (US and Canada) (4 studies), Asia East and Australia/New-Zealand (4 studies), Europe Centre/East/South (13 studies), Europe North (6 studies)

^c^Study designs were regrouped as follows: Observational cohort (3 studies), Retrospective cohort/chart-review/cross-sectional (9 studies), epidemiological survey (3 studies)

^d^Study designs were regrouped as follows: Cross-sectional (10 studies), Prospective cohort and survey (8 studies), Retrospective cohort/chart-review (9 studies)

^e^Total and residual between-study variance: the overall heterogeneity corresponds to the total between-study variance estimated from random-effects MA whereas the residual heterogeneity corresponds to between study-variance explained by the study-level covariates included into meta-regression model

^f^R^2^ is the proportion of the overall heterogeneity (i.e. the total between-study variance) which is “explained” (i.e. reduced) by the effect of the included study-level covariate

## Discussion

This systematic review provides an updated broad overview on the global epidemiology of DMD, including an evaluation of the quality of study reporting along with testing for publication bias. To our knowledge, this is the first comprehensive systematic review which evaluated the pooled global epidemiology of DMD. The pooled global prevalence and birth prevalence of DMD were 7.1 (95% CI: 5.0–10.1) and 19.8 (95% CI: 16.6–23.6) per 100,000 males, respectively. The birth prevalence is much higher than the prevalence because children with DMD may not survive beyond pediatric age likely in developing Countries with low adherence to standards of care. When considering as denominator the general population, the pooled global prevalence of DMD decreases, as expected, to 2.8 (95% CI: 1.6–4.6) cases per 100,000 as only males can be affected by the disease. Although epidemiological estimates were comparable in most studies, various outliers were found. The accuracy of these estimates could be strongly affected by different data sources (i.e. primary or secondary data), study design (e.g. prospective vs. retrospective studies, longitudinal vs. cross-sectional studies etc.), case definitions, inclusion criteria, sample sizes and DMD diagnostic methods, that could lead to extremely variable epidemiological estimations. In the present study we were not able to stratify results by ethnicity as this was not reported in all the studies; this might be important as it is known that some rare diseases such as Gaucher’s disease are known to be more common in specific ethnic groups, such as Ashkenazi Jews [69]. It was also not possible to compare the epidemiology across different countries, because of the small number of studies and large heterogeneity among the conducted studies.

Pooling the results of the different epidemiological studies, especially in the case of rare diseases, is particularly advantageous, since this increase of the total sample size allows more robust estimates and accounts for the potential differences among the included studies. Since the prevalence estimated within each included study was corroborated by a very small 95%CI (i.e. by a very small within-study variability or, in other words, by a very high precision) and since the I^2^ can be also expressed in terms of both the within-study variability (w) and the between-studies variability (b) components as follows: b/(w + b), it is clear that relatively small within-study variability will result in large I^2^ estimates (and this was the case) [21]. In response to this shortcoming, I^2^ estimates were also accompanied by their associated 95% CI [70, 71] because imprecise or biased estimates of heterogeneity can have serious consequences: for instance its overestimation may trigger inappropriate exploration of the cause(s) of heterogeneity. Nevertheless, such meta-analysis improves the accuracy and the reliability of the pooled estimate. The meta-regression analysis was useful to identify possible sources of heterogeneity by means of the use of study-level covariates. Interestingly, the only covariate which reduced the highest proportion of heterogeneity (about 45%) among DMD birth prevalence estimates was the year in which the study was carried out and its duration. The remaining heterogeneity could not be statistically accounted for.

Most studies included in this review were European, while only seven studies (15.9%) were identified from North America and no studies from South America were found. Overall, only 9 studies were found in Asia, Africa, Australia and New Zealand, all dated prior to 2005. Epidemiological research is essential to assess the population impact of rare diseases and to support public health decision-making: while epidemiological research can inform and improve public policy, public policy can also encourage and support epidemiological research. In Europe, rare diseases are among the priorities in public health research identified by the European Commission as of 2007 through FP7 programs and later through Horizon 2020 funding programs [72]. It may not be a coincidence that a review on public policy conducted in 2018 suggested that the European countries presented the most unified approach to rare diseases, while no rare disease policies were found in Africa, India and Russia [73].

The majority of the studies included used real-world data sources, such as claims databases, electronic medical records (EMRs) and patient/disease registries. Such data sources have a significant, and often under-used, potential to study rare diseases and to carry out accurate epidemiological evaluations [74]. The main advantage of using real-world data sources is the size of the catchment population, which is often very large, in the order of millions [75]. While this is an advantage in any research setting, it is particularly valuable to study rare diseases because the incidence of these diseases is so low.

However, there are also limitations to using each specific type of secondary data such as those from claims databases, EMRs and/or registries. One of the principal obstacles in using these data sources to study rare diseases is related to disease coding through systems such as International Classification of Diseases, 9th Revision (ICD-9), International Classification of Diseases, 10th Revision (ICD-10) and so on. While this is most relevant for claims and EHRs, some registries may also use ICD or similar codes [74].

Rare diseases commonly do not have a medical code specific to them. Taking DMD as an example, the ICD-9 code refers to muscular dystrophy in general, which includes but is not limited to DMD (ICD9-CM code: 359.1) [74]. Similarly, the ICD-10 code closest to DMD includes DMD but is not specific to it as it refers to Duchenne or Becker’s muscular dystrophy (ICD-10 code: G71.01). As a result, the specificity of the diagnosis in data systems that use these codes is not very high in DMD and in rare diseases with similar issues. One solution to this problem might be linking claims databases/electronic medical records to registers of rare diseases from the same catchment area, whenever available, to validate DMD diagnoses recorded in claims databases by comparing them to the gold standard diagnosis, i.e. the diagnosis in patient registers [74]. This approach was followed in the study conducted by König et al. [68], where DMD patients where identified through the linkage of clinical records and patient registers. However, even this approach has its limitations: the DMD prevalence measured in the study conducted by König et al. fell significantly (from 11.7 per 100,000 in 2014 to 1.5 per 100,000 in 2017) in the last few years of the study due to missing data as a result of privacy issues.

The role of patient registers in the published literature has been acknowledged as an important real-world data source on rare diseases for many years, although they have been underused because of barriers to data access. Registers provide a unique opportunity to follow the natural history of the disease in time [76]. The main limitation of registers with regards to the epidemiology of a rare disease is that the catchment area and its population (i.e. the denominator, whether in persons or person-years) may not be clearly defined. This would make it difficult to estimate the frequency of the diagnosis being made.

Apart from disease coding, another common tool for DMD identification in the studies included in the present review was genetic testing. Genetic testing for DMD is arguably the most reliable method of identifying DMD patients. There are at least three types of genetic tests available for DMD to date, i.e. tests for genetic duplications or deletions, CGH-array and direct sequencing. However, it is likely that quality of these tests increased over time. As a result, the reliability of DMD identification in earlier studies may not be as accurate as more recently conducted studies. In general, the identification of DMD patients in secondary data sources based on a diagnosis which is not directly associated with a genetic test is likely to be a less valid method than an identification method which is based primarily on genetic testing. However, the more accurate identification of true cases, for example, by genetic testing, does not necessarily lead to more accurate epidemiological estimates. Two studies which both used genetic testing to identify DMD reported much a higher prevalence per 100,000 males than the pooled estimate and were not consistent with other studies: Darin et al. [36] who reported a prevalence of 16.8 (95% CI: 11.8–23.8) and Rasmussen et al. [43], who reported a prevalence of 16.2 (95% CI: 11.4–22.9). The common elements between these two studies are the relatively low number of cases and the low number of persons in the source population, compared to other studies reporting the prevalence. These studies are more prone to over- or under-estimate the true number of cases based on a small sample size, even though they used genetic testing to identify DMD. This could in turn contribute to heterogeneity.

The overall quality of the studies, which reflects the transparency of reporting, included in the present review was assessed using a checklist adapted from STROBE. The results of the assessment suggest that the overall quality of study reporting was medium to low. In particular, although the majority of the studies adequately described the study design and setting, most of them did not report the eligibility criteria or an adequate characterization of the study participants (e.g. mean age, ethnicity). In some cases, this was in line with the research question of the studies, which did not address DMD alone but with other dystrophies [26–29, 32–36, 38, 40–46, 53, 56, 59, 61, 68]. Future studies should address the clinical picture of DMD patients on a large scale, as this is very informative concerning several aspects such as unmet clinical needs, overall survival and cost of care. The quality of reporting and the transparency in how the research was carried out are important because they impact how useful the study is [77]. This has been highlighted for observational research in epidemiology in general, but may be even more important for rare diseases, since the manner in which data is collected and the data analysis is carried out can potentially lead to a very large variability of results due to the very small sample size. An additional problem that follows is that it becomes very difficult to replicate studies. From the present paper, it is clear that the transparency of reporting of observational studies concerning DMD needs to improve significantly.

### Strengths and limitations

The main strengths of our systematic review and meta-analysis are the exhaustive literature search strategy and the double review process as well as the inclusion of studies published very recently. Meta-regression analysis is another strength of this study, which allowed us to identify the main drivers of heterogeneity. Moreover, we restricted the meta-analysis for medium quality studies, in order to have an estimate that is not affected by low-quality studies. Nevertheless, the stratified meta-analysis was in line with the main results.

However, several limitations should be considered. We have tried to describe the studies in as much detail as possible, including the heterogeneity among them. The quality of our analyses could be affected by the intrinsic limitations of each included article and the different DMD outcome definitions of the individual studies could compromise the internal validity of this meta-analysis. Furthermore, although no publication bias was found, the between-studies heterogeneity was very high. Moreover, we were not able to stratify our results by ethnicity as this was not reported in all the studies.

## Conclusion

To our knowledge, this is the first systematic review to evaluate the pooled global epidemiology of DMD and to assess the quality of study reporting. Due to the wide differences between each study (e.g. study design and setting, study population, data sources, case ascertainment, etc.) DMD prevalence and birth prevalence estimates are variable throughout the literature, ranging 0.9 to 16.8 per 100,000 males from 1.5 to 28.2 per 100,000 live male births, respectively. The pooled prevalence and birth prevalence were 5.3 (95% CI: 5.1–5.5) cases per 100,000 males and 21.4 (95% CI: 20.4–22.5) cases per 100,000 live male births respectively. Generating epidemiological evidence on DMD is fundamental to support public health decision-making in allocating resources considering the high disease’s costs related to the need of multidisciplinary care, the elevated direct and indirect burden of patients and caregivers and the recently available expensive therapies. The overall quality of epidemiological studies on DMD was relatively low, highlighting the need for high quality studies in this field. High quality studies with more transparent reporting are required to better understand the epidemiology of DMD.

## Supplementary information

**Additional file 1.** Preferred Reporting Items for Systematic Reviews and Meta-Analyses (PRISMA) checklist.

**Additional file 2.** Literature search strategies.

**Additional file 3.** Adapted checklist for reporting items in observational studies of rare diseases (adapted from strobe checklist) – taken from Leady et al., 2014 (DOI: https://doi.org/10.1186/s13023-014-0173-x).

**Additional file 4.** Quality of study reporting assessment.

## Data Availability

Systematic review and meta-analysis performed on already published papers. The databases used to conduct this study and the PRISMA flow diagram are included in the main text and the detailed literature search strategy is reported in the supplementary materials.

## Abbreviations

DMD: Duchenne Muscular Dystrophy
STROBE: STrengthening the Reporting of OBservational studies in Epidemiology
CK: Creatinine kinase
DBMD: Duchenne/Becker muscular dystrophy
95% CI: 95% confidence interval
ADHD: Attention Deficit Hyperactivity Disorder
PRISMA: Preferred Reporting Items for Systematic Reviews and Meta-Analyses
SE: Standard error
I^2^: Inconsistency
EMRs: Electronic medical records
ICD-8: International Statistical Classification of Diseases and Related Health Problems, 8th edition
ICD-9: International Classification of Diseases, 9th Revision
ICD-10: International Classification of Diseases, 10th Revision
MPLA: Multiplex ligation-dependent probe amplification
df: Degrees of freedom
MA: Meta-analysis
