# Supplementary material for: Global epidemiology of Duchenne muscular dystrophy: an updated systematic review and meta-analysis
Source: Orphanet J Rare Dis. 2020 Jun 5;15:141. doi: 10.1186/s13023-020-01430-8 (PMC7275323; doi:10.1186/s13023-020-01430-8)

**Additional file 1.** Preferred Reporting Items for Systematic Reviews and Meta-Analyses (PRISMA) checklist


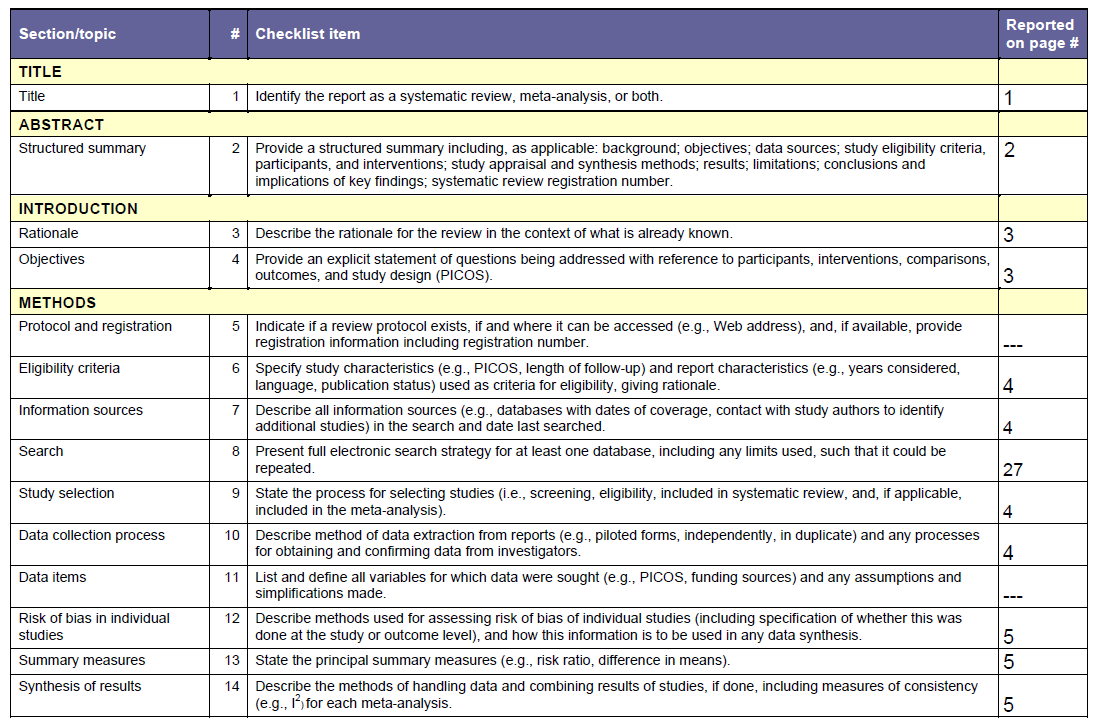


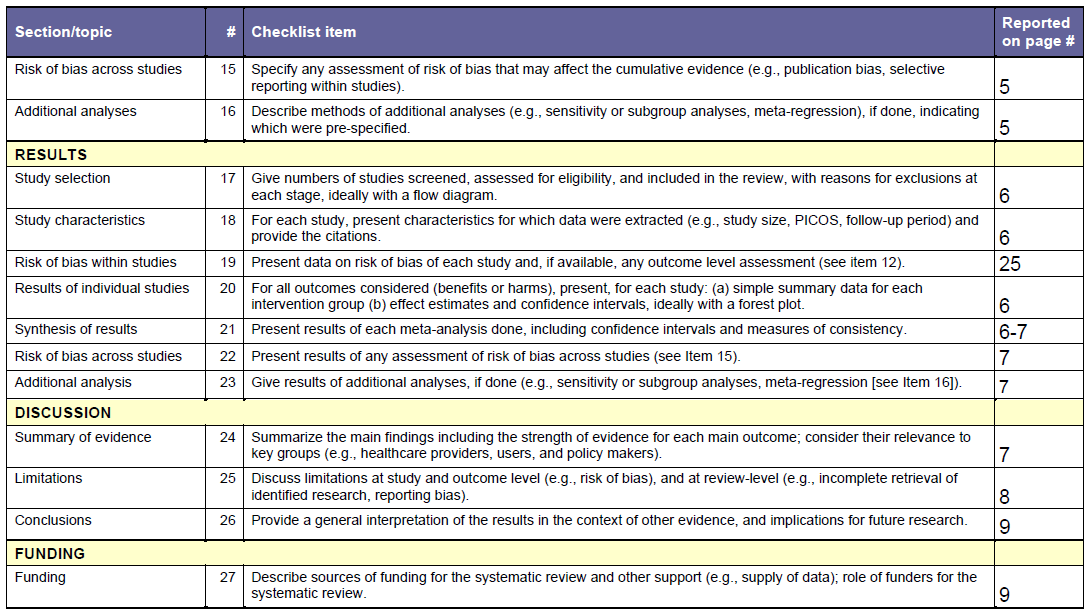

Supplement: Supplementary file 1 — Additional file 1. Preferred Reporting Items for Systematic Reviews and Meta-Analyses (PRISMA) checklist. [file 13023_2020_1430_MOESM1_ESM.docx]
