# Supplementary material for: Global epidemiology of Duchenne muscular dystrophy: an updated systematic review and meta-analysis
Source: Orphanet J Rare Dis. 2020 Jun 5;15:141. doi: 10.1186/s13023-020-01430-8 (PMC7275323; doi:10.1186/s13023-020-01430-8)
Supplement: Supplementary file 2 — Additional file 2. Literature search strategies. [file 13023_2020_1430_MOESM2_ESM.docx]

**Additional file 2.** Literature search strategies

Database: MEDLINE

1. Incidence/ 2,795,270

2. Prevalence/ 2,631,627

3. Epidemiolog*/ 2,007,830

4. Duchenne Muscular Dystrophy/ 11,322

5. #1 or #2 or #3/ 3,156,889

6. #4 and #5/ 773

Database: EMBASE

1. exp Incidence/ 1,164,339

2. exp Prevalence/ 997,549

3. Epidemiolog*/ 2,055,058

4. #1 or #2 or #3/ 3,459,715

5. #4 and #5/ 1,178
