# Supplementary material for: Global epidemiology of Duchenne muscular dystrophy: an updated systematic review and meta-analysis
Source: Orphanet J Rare Dis. 2020 Jun 5;15:141. doi: 10.1186/s13023-020-01430-8 (PMC7275323; doi:10.1186/s13023-020-01430-8)
Supplement: Supplementary file 3 — Additional file 3. Adapted checklist for reporting items in observational studies of rare diseases (adapted from strobe checklist) – taken from Leady et al., 2014 (DOI: https://doi.org/10.1186/s13023-014-0173-x). [file 13023_2020_1430_MOESM3_ESM.docx]

**Additional file 3.** Adapted checklist for reporting items in observational studies of rare diseases (adapted from strobe checklist) – taken from Leady et al., 2014 (DOI: 10.1186/s13023-014-0173-x).

1. **Was there an adequate description of study design and setting?**

**YES** if:

- Paper described the method of data collection (e.g. retrospective epidemiological survey, records from a list of sources)
- Paper described the setting (e.g. clinics, population registered at general practices, medical records database)
- Paper relevant dates (periods for recruitment, data collection).
- Paper give the source of denominator population for prevalence calculations (e.g. UK national statistics)

**NO** if paper did not report all of the above

**UNCLEAR** if paper reported design and setting information but it was presented unclearly or incompletely (e.g. the number of general practices was not reported or only the recruitment start date was reported)

1. **Was there an adequate description of eligibility criteria?**

**YES** if**:**

- Paper described inclusion criteria (exclusion criteria are not necessary)
- Paper explicitly stated which type of MPS IV they report (specifically MPS IVA or MPS IVB)
- Paper include enzymatic or genetic analysis as diagnoses methods (it is sufficient to state patients were enzymatically diagnosed without giving full details)

**NO** if paper did not report all of the above

**UNCLEAR** if**:**

- Paper reported eligibility criteria but it was presented unclearly (if MPS IVA is reported but no diagnostic method)
- Paper did not clearly state which type of MPS IV was reported

1. **Is the study population representative of the target population?**

Note – for this question, the target population is the population studied in the study, not the population that we are studying for this systematic review. Ethnicity is not important, as long as the patient lives in the given country.

**YES** if paper states the sources include all necessary diagnostic centres or that they have attempted to achieve full ascertainment or have outlined an extensive list of sources

**NO** if there is reason to believe that full ascertainment has not been achieved

**UNCLEAR** if we cannot be sure that all patients were included in the study (e.g. in a country multiple centres could have performed the diagnostic analyses and not all participated in the study).

1. **Is there an adequate description of outcomes?**

**YES** if**:**

- Paper describes patients in denominator – live births or general population
- Paper describes patients in numerator were born during study period (birth prevalence) or were living during study period (period prevalence)
- Paper describes time frame of study
- Paper describes the period of study e.g. ‘date of first diagnosed’ case to last diagnosed case or ‘date of birth of first diagnosed case’ to last diagnosed case

**NO** if paper did not report all of the above

**UNCLEAR** if any of the above are not clearly reported

1. **Is there an adequate description of the study participants?**

**YES** if the paper provided more than just age (at diagnosis) and gender (for example ethnicity) then I would say the participants were adequately described

**NO** if paper did not report more than age and gender

**UNCLEAR** if the population descriptions were unclear (e.g. numbers in texts and figures didn’t match or add up).

**Overall score:**

High – all criteria met (5 Yes’s)

Medium – 1 to 2 criteria not met (i.e. 1-2 No’s or Unclear)

Low – 3 or more criteria not met (i.e. ≥ 3 No’s or Unclear)
