# Supplementary material for: Global epidemiology of Duchenne muscular dystrophy: an updated systematic review and meta-analysis
Source: Orphanet J Rare Dis. 2020 Jun 5;15:141. doi: 10.1186/s13023-020-01430-8 (PMC7275323; doi:10.1186/s13023-020-01430-8)
Supplement: Supplementary file 4 — Additional file 4. Quality of study reporting assessment. [file 13023_2020_1430_MOESM4_ESM.docx]

**Additional file 4**. Quality of study reporting assessment

| First author and publication year | Country | 1. Was there an adequate description of study design and setting? | 2. Was there an adequate description of eligibility criteria? | 3. Is the study population representative of the target population? | 4. Is there an adequate description of outcomes? | 5. Is there an adequate description of the study participants? | Overall assessment |
| --- | --- | --- | --- | --- | --- | --- | --- |
| Ahlström, 1993 | Sweden | Yes | Yes | Yes | Yes | No | Medium |
| Ballo, 1994 | South Africa | Yes | Unclear | Yes | Yes | Yes | Medium |
| Bertolotto, 1981 | Turin, Italy | Yes | Yes | Yes | Yes | No | Medium |
| Bradley, 1993 | Wales | Yes | No | Yes | Yes | No | Medium |
| Brooks, 1977 | South Eastern Scotland | Yes | No | Yes | Yes | No | Medium |
| Chung, 2003 | Hong Kong | Yes | Yes | Yes | Yes | No | Medium |
| Cowan, 1980 | Australia | Yes | No | Yes | Yes | No | Medium |
| Danieli, 1977 | Veneto, Italy | Yes | Unclear | Yes | Yes | No | Medium |
| Danieli, 1980 | Veneto, Italy | Yes | Yes | Yes | Yes | No | Medium |
| Darin, 2000 | Western Sweden | Yes | Yes | Yes | Yes | No | Medium |
| Dellamonica, 1983 | France | Unclear | Yes | Yes | Yes | No | Medium |
| Dooley, 2010 | Nova Scotia, Canada | Yes | No | Yes | Yes | No | Medium |
| Drousiotou, 1998 | Cyprus | Yes | No | Yes | Yes | No | Medium |
| Drummond, 1979 | New Zealand | No | No | No | Yes | No | Low |
| El-Tallawy, 2005 | Assiut, Egypt | Yes | No | Yes | Yes | Yes | Medium |
| Eyskens, 2006 | Antwerp, Belgium | Yes | No | Yes | Yes | No | Medium |
| Greenberg, 1988 | Canada | Yes | No | Yes | Yes | No | Medium |
| Hughes, 1996 | Northern Ireland | Yes | No | Unclear | Yes | No | Low |
| Jeppesen, 2003 | Aarhus, Denmark | Yes | Yes | Yes | Yes | No | Medium |
| König, 2019 | Germany | Yes | No | Yes | Yes | No | Medium |
| Lefter, 2016 | Republic of Ireland | Unclear | Yes | Yes | Unclear | No | Low |
| Leth, 1985 | Denmark | No | Unclear | Yes | Unclear | No | Low |
| Mah, 2011 | Canada | Yes | No | Unclear | Unclear | No | Low |
| Mendell, 2012 | Ohio, USA | Yes | Yes | Yes | Yes | No | Medium |
| Merlini, 1992 | Bologna, Italy | Yes | Unclear | Yes | Yes | No | Medium |
| Moat, 2013 | Wales | Yes | No | Yes | Yes | No | Medium |
| Monckton, 1982 | Alberta, Canada | Yes | Yes | Unclear | Yes | No | Medium |
| Mostacciuolo, 1987 | Veneto, Italy | Yes | Yes | Yes | Yes | No | Medium |
| Nakagawa, 1991 | Okinawa, Japan | Yes | Yes | Yes | Unclear | No | Medium |
| Nigro, 1983 | Campania, Italy | Yes | Yes | Yes | Yes | No | Medium |
| Norman, 1989 | Wales | No | Yes | Yes | No | No | Low |
| Norwood, 2009 | Northern England | Yes | Yes | Yes | Yes | No | Medium |
| Peterlin, 1997 | Slovenia | Yes | Yes | Yes | Yes | No | Medium |
| Radhakrishnan, 1987 | Benghazi, Libya | Unclear | Yes | Yes | No | No | Low |
| Ramos, 2016 | Puerto Rico | Yes | Yes | Yes | Yes | No | Medium |
| Rasmussen, 2012 | South-Eastern Norway | Yes | Yes | Yes | Yes | No | Medium |
| Romitti, 2015 | United States of America | Yes | Unclear | Yes | Yes | No | Medium |
| Scheuerbrandt, 1986 | West Germany | No | No | Unclear | Yes | No | Low |
| Siciliano, 1999 | Tuscany, Italy | Yes | Yes | Yes | Unclear | Yes | Medium |
| Takeshita, 1977 | Shimane, Japan | Yes | No | Yes | Yes | No | Medium |
| Takeshita, 1987 | Japan | Yes | Yes | Unclear | Yes | No | Medium |
| Talkop, 2003 | Estonia | Yes | Yes | Unclear | Yes | Yes | Medium |
| Tangsrud, 1989 | Southern Norway | Yes | Yes | Yes | Yes | No | Medium |
| van Essen, 1992 | The Netherlands | Yes | Yes | Yes | Yes | No | Medium |
